# Supplementary figures and images for: A recombinant gp145 Env glycoprotein from HIV-1 expressed in two different cell lines: Effects on glycosylation and antigenicity
Source: PLoS One. 2020 Jun 19;15(6):e0231679. doi: 10.1371/journal.pone.0231679 (PMC7304579; doi:10.1371/journal.pone.0231679)

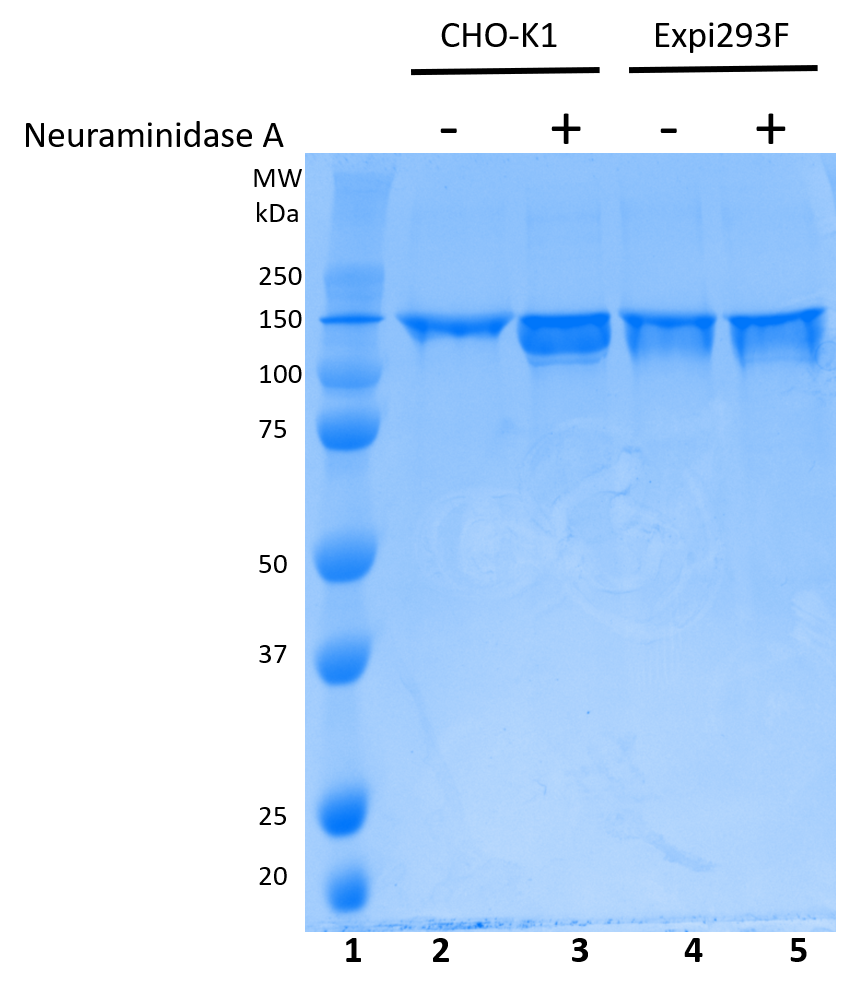

Supplement: S1 Fig — The recombinant gp145 immunogens were incubated overnight with (+, lanes 3 and 5) and without (-, lanes 2 and 4) Neuraminidase A, and then resolved on 10% SDS-PAGE gels. (TIF) [file pone.0231679.s001.tif]

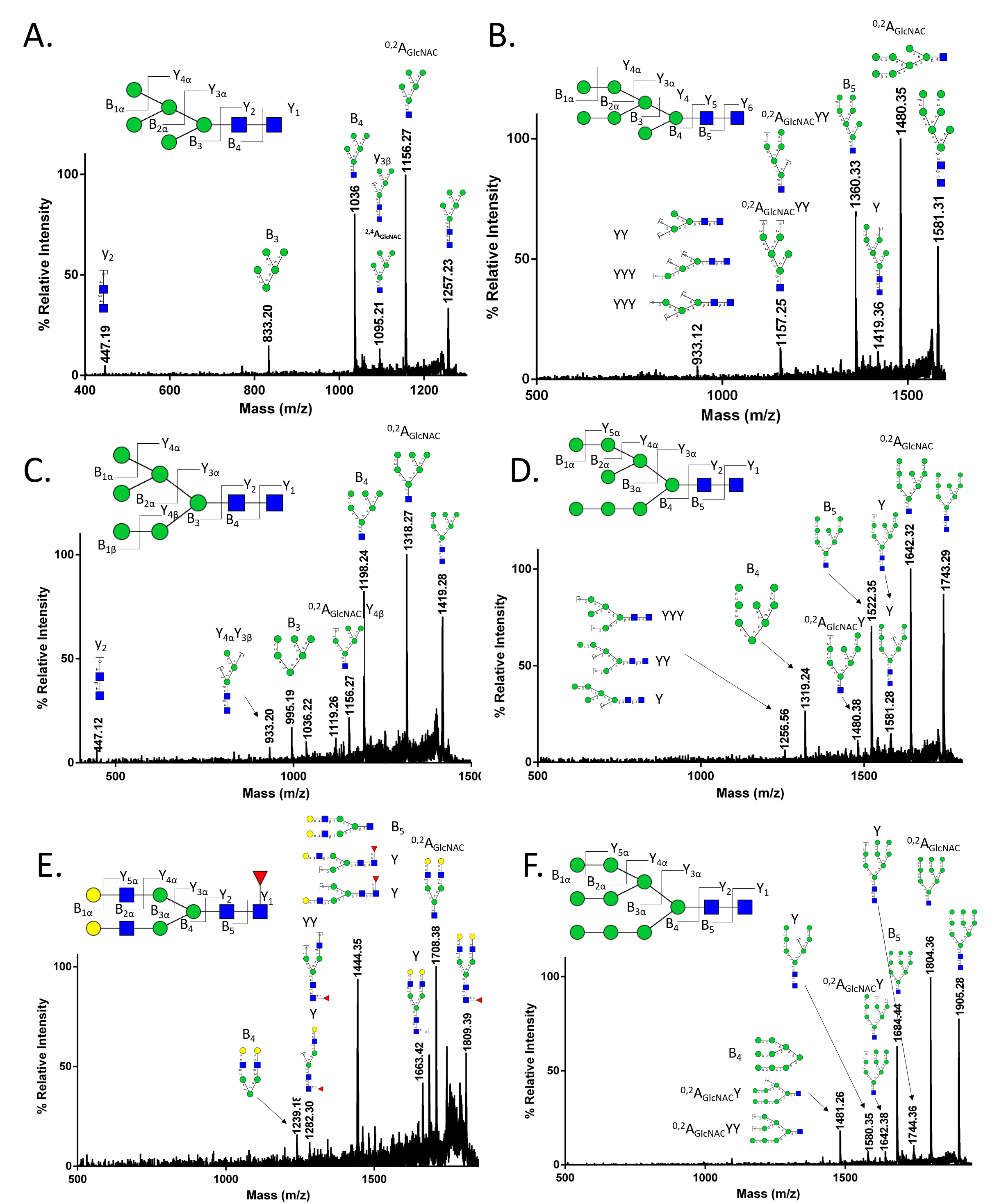

Supplement: S2 Fig — The presence of Man5GlcNAc2 (A), Man6GlcNAc2 (B), Man7GlcNAc2 (C), Man8GlcNAc2 (D), Gal2Man3GlcNAc4Fuc1 (E) and Man9GlcNAc2 (F) was confirmed by MALDI-PSD. The x-axis represents the mass-to charge ratio (m/z) value in Daltons and the y-axis shows the relative abundance (arbitrary units) of the ions. (TIF) [file pone.0231679.s002.tif]
