# Supplementary material for: A recombinant gp145 Env glycoprotein from HIV-1 expressed in two different cell lines: Effects on glycosylation and antigenicity
Source: PLoS One. 2020 Jun 19;15(6):e0231679. doi: 10.1371/journal.pone.0231679 (PMC7304579; doi:10.1371/journal.pone.0231679)
Supplement: S1 Table — All the peaks obtained in the MALDI-ToF were compared against the list previously reported by Doores et al., 2010. Some of them were selected randomly for confirmation by MS/MS analysis. (DOCX) [file pone.0231679.s003.docx]

| **Supplementary Table 1. *N*-glycans composition of C06980v0c22 gp145 using MALDI-ToF in Reflector Positive Ion Mode**  **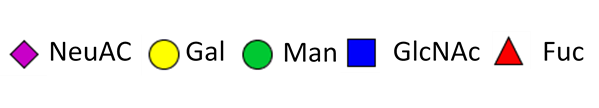** | | | | |
| --- | --- | --- | --- | --- |
| ***m/z*** | | | **Predicted Structure** | **Ions** |
| **Observed** | | **Calculated** |  |  |
| **CHO-K1 gp145** | **Expi293F gp145** |  |  |  |
| 1257.41 | 1257.47 | 1257.42 | 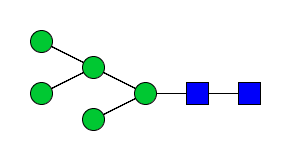 | [M + Na]^+^ |
| 1298.43 | 1298.49 | 1298.45 | 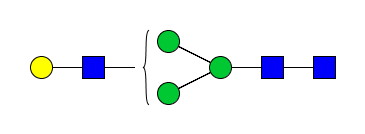 | [M + Na]^+^ |
| 1419.45 | 1419.51 | 1419.48 | 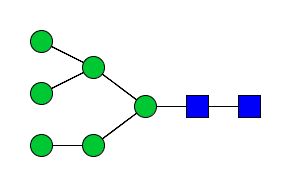 | [M + Na]^+^ |
| 1444.48 | 1444.54 | 1444.51 | 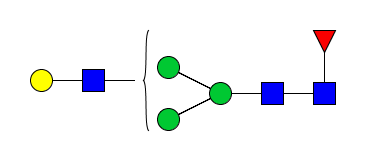 | [M + Na]^+^ |
| 1460.46 | 1460.52 | 1460.50 | 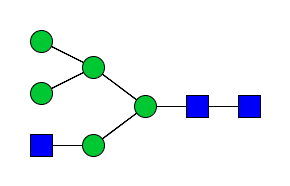  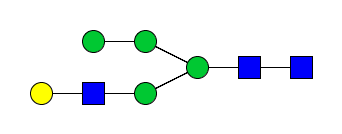 | [M + Na]^+^ |
| 1480.45 | 1489.40 | 1479.55 | 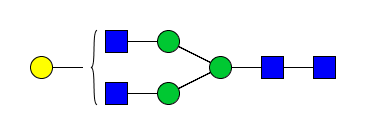  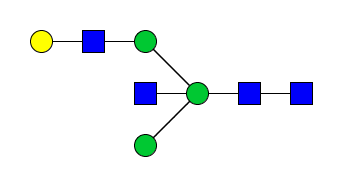 | [M + H]^+^ |
| - | 1485.55 | 1485.53 | 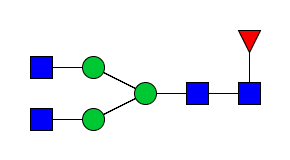 | [M + Na]^+^ |
| 1502.48 | 1502.55 | 1501.53 | 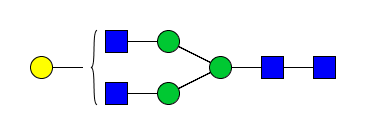 | [M + Na]^+^ |
| 1581.49 | 1581.56 | 1581.53 | 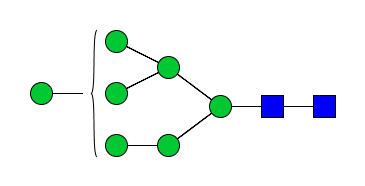 | [M + Na]^+^ |
| - | 1606.68 | 1606.56 | 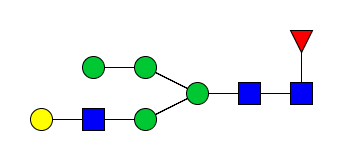  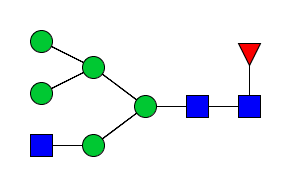 | [M + Na]^+^ |
| 1622.52 | 1622.59 | 1622.55 | 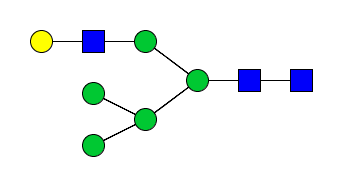 | [M + Na]^+^ |
| 1642.45 | 1642.50 | 1641.60 | 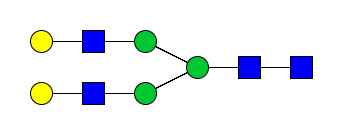  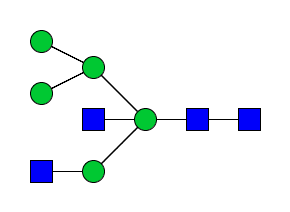 | [M + Na]^+^ |
| 1647.54 | 1647.61 | 1647.59 | 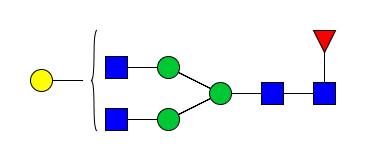 | [M + Na]^+^ |
| 1663.55 | 1663.62 | 1663.58 | 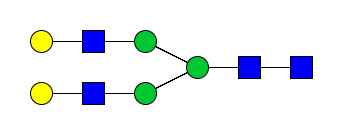 | [M + Na]^+^ |
| - | 1688.63 | 1688.61 | 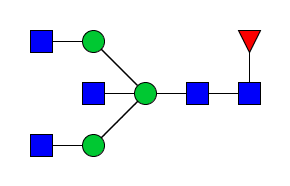 | [M + Na]^+^ |
| 1743.53 | 1743.61 | 1743.48 | 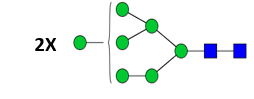 | [M + Na]^+^ |
| 1809.57 | 1809.64 | 1809.64 | 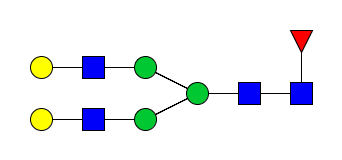 | [M + Na]^+^ |
| - | 1850.62 | 1850.67 | 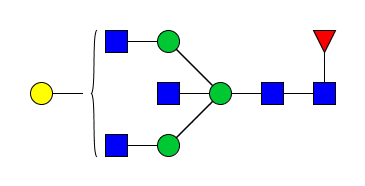 | [M + Na]^+^ |
| 1905.57 | 1905.65 | 1905.63 | 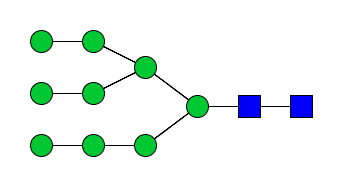 | [M + Na]^+^ |
| 2012.64 | 2012.71 | 2012.72 | 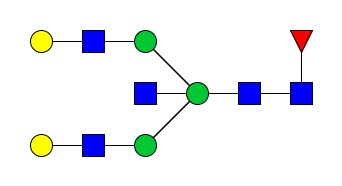 | [M + Na]^+^ |
| 2028.66 | 2028.71 | 2028.71 | 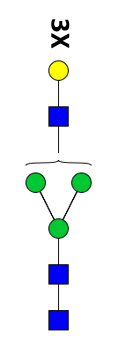 | [M + Na]^+^ |
| 2122.62 | 2122.67 | 2122.72 | 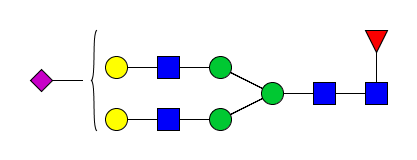 | [M–H+2Na]^+^ |
| 2174.68 | 2174.76 | 2174.68 | 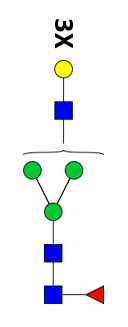 | [M + Na]^+^ |
| - | 2215.79 | 2215.80 | 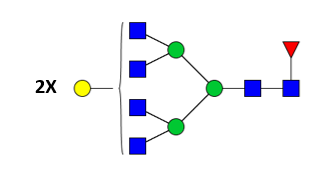 | [M + Na]^+^ |
| - | 2377.82 | 2377.85 | 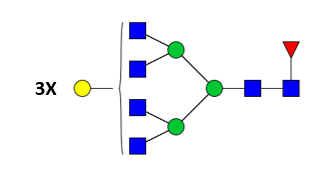 | [M + Na]^+^ |
| 2539.77 | 2539.86 | 2539.77 | 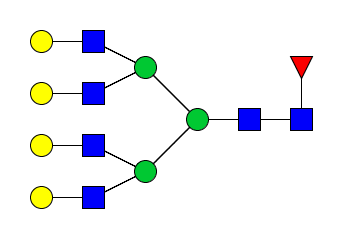 | [M + Na]^+^ |
