# Supplementary material for: A recombinant gp145 Env glycoprotein from HIV-1 expressed in two different cell lines: Effects on glycosylation and antigenicity
Source: PLoS One. 2020 Jun 19;15(6):e0231679. doi: 10.1371/journal.pone.0231679 (PMC7304579; doi:10.1371/journal.pone.0231679)
Supplement: S2 Table — (DOCX) [file pone.0231679.s004.docx]

| **Supplementary Table 2. *N*-glycans composition of C06980v0c22 gp145 using MALDI-ToF in Reflector Negative Ion Mode**  **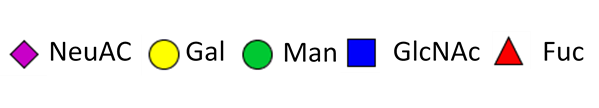** | | | | |
| --- | --- | --- | --- | --- |
| ***m/z*** | | | **Predicted Structure** | **Ions** |
| **Observed** | | **Calculated** |  |  |
| **CHO-K1 gp145** | **Expi293F gp145** |  |  |  |
| 1565.43 | - | 1565.55 | 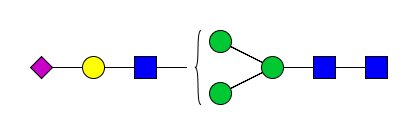 | [M - H]^-^ |
| 1718.43 | 1719.46 | 1719.58 | 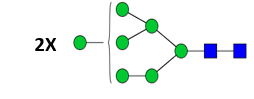 | [M - H]^-^ |
| 1880.47 | 1881.53 | 1881.64 | 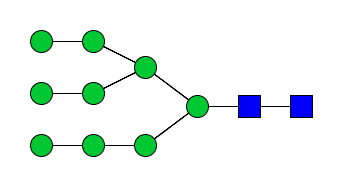 | [M - H]^-^ |
| 1930.50 | 1930.52 | 1930.68 | 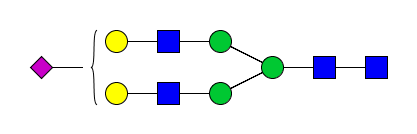 | [M - H]^-^ |
| 2076.54 | 2076.58 | 2076.74 | 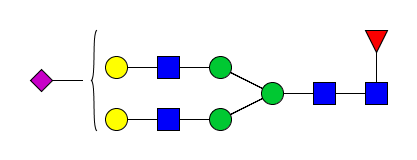 | [M - H]^-^ |
| 2118.50 | 2118.54 | 2117.76 | 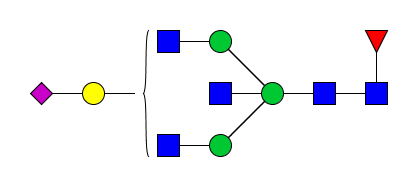 | [M - H]^-^ |
| 2238.43 | - | 2238.79 | 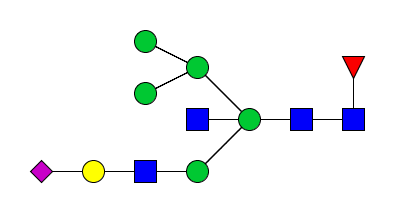 | [M - H]^-^ |
| 2367.59 | - | 2367.83 | 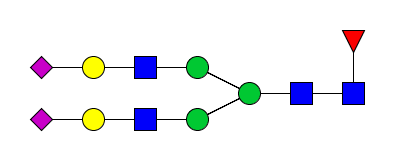 | [M - H]^-^ |
| 2389.56 | 2389.58 | 2389.82 | 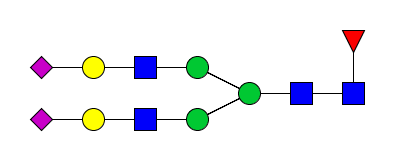 | [M –2H+Na]^-^ |
| 2441.60 | 2441.60 | 2441.87 | 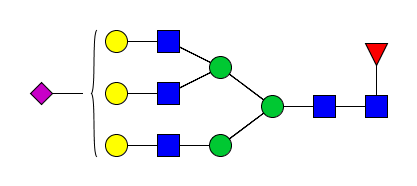 | [M - H]^-^ |
| 2754.62 | 2754.65 | 2754.95 | 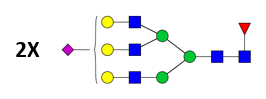 | [M - H]^-^ |
| - | 2807.63 | 2807.00 | 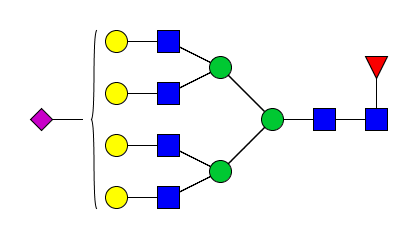 | [M - H]^-^ |
