# Supplementary material for: A recombinant gp145 Env glycoprotein from HIV-1 expressed in two different cell lines: Effects on glycosylation and antigenicity
Source: PLoS One. 2020 Jun 19;15(6):e0231679. doi: 10.1371/journal.pone.0231679 (PMC7304579; doi:10.1371/journal.pone.0231679)
Supplement: S3 Table — Binding kinetic results were obtained using the Octet Red 96 system. Biotinylated gp145 was immobilized on streptavidin sensors, dipped into two-fold dilutions of the monoclonal antibodies and association/dissociation rates were analyzed using the Octet Molecular Interaction System software. *All CHO-K1 antigenicity results were previously reported by Wieczorek et al. 2015 (16). (DOCX) [file pone.0231679.s005.docx]

| **Supplementary Table 3. Binding of CO6980v0c22 gp145 to HIV-1 antibodies** | | | | | |
| --- | --- | --- | --- | --- | --- |
| **Antibody** | **Specificity** | **gp145 Expression System*** | **Ka (1/Ms)** | **Kd (1/s)** | **KD (nM)** |
| CD4 | CD4bs | CHO-K1 | 1.36E+05 | 7.533E-04 | 5.53 |
|  |  | Expi293F | 2.10E+05 | 2.60E-04 | 1.2 |
| b6 | CD4bs non NE | CHO-K1 | 3.52E+05 | 5.76E-06 | <0.016 |
|  |  | Expi293F | 5.40E+05 | <1.0E-07 | <1. 0E-03 |
| VRC01 | CD4bs NE | CHO-K1 | 6.22E+04 | 9.26E-06 | 0.149 |
|  |  | Expi293F | 5.60E+04 | <1.0E-07 | <1. 0E-03 |
| PG9 | V1/V2 NE | CHO-K1 | 4.74E+04 | 2.64E-04 | 5.6 |
|  |  | Expi293F | 1.00E+05 | 2.51E-04 | 2.1 |
| 2158 | V2 (α4β7bs) | CHO-K1 | 3.20E+04 | 6.50E-06 | 0.2 |
|  |  | Expi293F | 3.40E+04 | <1.0E-07 | <1.0E-03 |
| 447-52D | V3 | CHO-K1 | 1.03E+06 | 1.02E-02 | 9.9 |
|  |  | Expi293F | 1.08E+06 | 7.98E-03 | 7.4 |
| PGT121 | V1/V2 and V3 Glycan | CHO-K1 | 1.52E+04 | 1.42E-03 | 93.3 |
|  |  | Expi293F | 1.77E+04 | 7.18E-04 | 40.6 |
| 4E10 | MPER | CHO-K1 | 2.23E+04 | 2.09E-03 | 27.1 |
|  |  | Expi293F | 1.33E+04 | 2.22E-04 | 16.7 |
