# Supplementary material for: A recombinant gp145 Env glycoprotein from HIV-1 expressed in two different cell lines: Effects on glycosylation and antigenicity
Source: PLoS One. 2020 Jun 19;15(6):e0231679. doi: 10.1371/journal.pone.0231679 (PMC7304579; doi:10.1371/journal.pone.0231679)
Supplement: Attachment — Submitted filename: Response-Letter-3-PONE-D-20-07724.doc [file pone.0231679.s007.doc]

**UNIVERSITY OF PUERTO RICO, UNIVERSIDAD DE PUERTO RICO**

**MEDICAL SCIENCES CAMPUS, RECINTO DE CIENCIAS MEDICAS**

**SCHOOL OF MEDICINE, ESCUELA DE MEDICINA**

**DEPARTMENT OF BIOCHEMISTRY**

**DEPARTAMENTO DE BIOQUIMICA**

**Response to Reviewer #1:** Thank you for your input, which made this manuscript better and more polished.

**Response to Reviewer #2:** We greatly appreciate your insightful comments on the suitability of an uncleaved gp145 as a protective immunogen. Your feedback has given us the chance to reflect on our efforts to produce viral glycoproteins and to discuss possible avenues for the production of protein-based vaccines against SARS-CoV-2.

*“The author’s explanation of why they used an uncleaved clade C gp145 is satisfactory. These reasons should be stated clearly in the paper to provide proper context to anchor a reasons as to why the work was done - that the product is in a clinical trial so it is useful to know this information. As it stood, there was no explanation for the choice of strain and format. I think the point that it is in clinical trial should even be stated in the abstract, as I really think that will increase reader’s interest which might otherwise be lost due to the uncleaved platform.”*

We have included a sentence in the abstract and in the introduction to mention the on-going clinical trial, as suggested by the reviewer. And we do agree with the reviewer that the fact CO6980v0c22 is being tested clinically, makes it more relevant to the average reader. However, the objective of our manuscript is to compare biochemical properties for this immunogen expressed in CHO and 293 cells. We are not making any claims of efficacy or immunogenicity for this specific construct. The observations reported by us would still be valid if the gp145 immunogen did not elicit the desired neutralizing humoral response in humans, and could still be useful to researchers expressing other viral glycoproteins for purposes other than vaccine development.

“*I don’t believe that uncleaved gp145 is a useful format, regardless of the clinical trial. At least from the perspective of inducing neutralizing antibodies, uncleaved gp140, like gp120 monomer have by now been well-established in dozens of studies (antigenicity and immunogenicity) as poor choices.* *It may be in both of the clinical trials mentioned in the response letter that neutralizing antibodies are not the end goal and that cellular or non-neutralizing antibodies are instead the goals. If this is the case, however, evaluating the constructs with PG9 and 2G12 is not crucial and they could be assessed with other antibodies that don’t neutralize, like V3.”*

The description of the clinical trial (<https://clinicaltrials.gov/ct2/show/NCT03382418>) clearly states that among the secondary outcomes that will be measured are the response rates and levels of CD8+ and CD4+ T cells, and the response rates and levels of neutralizing antibodies. While the results of these measurements are not presently available, we have chosen not to speculate on what these results might eventually be. We do understand that the reviewer feels strongly that this vaccine is unlikely to yield a neutralizing response. Our manuscript, however, does not make any claims of efficacy or protection, as we focus on the differences in the chemical properties of the immunogen which may result from the choice of expression host.

*“The statement that gp120 doesn’t work line 61 and “longer” constructs are needed including MPER is a bit vague. This is not about counting epitopes and including them, but more about conformation. It makes more sense to state that forms that better resemble viral spikes are preferable. Uncleaved gp140 is not really much of an improvement on gp120 as it consists of loose gp120 protomers held together by hydrophobic gp41, as shown by EM.”*

Line 61: We do not disagree with this observation and have deleted the phrase: “to preserve known bNAb epitopes within the membrane proximal external region (MPER)” and inserted “that better resemble viral spikes”.

*“Overall, I would think that some acknowledgement that gp145 is not a cutting-edge platform for inducing neutralizing antibodies should be added somewhere, as otherwise the paper ignores much of the advances in vaccines in preclinical work in recent years. Uncleaved gp145 may have been a reasonable choice in say 2005, but not really in 2020. All this said, given that this product is in trial justifies the work, but the link to a trial is needed to be stated prominently to justify it.”*

We have included a sentence in the abstract and another one in the introduction to mention the on-going clinical trial, as suggested by the reviewer.

*“Regarding the point in the response letter that uncleaved trimers contain more mannose than SOSIP or membrane versions (PMID 26311893 and 26018173), this statement is at odds with the work of Pritchard mentioned directly above this in the letter where there are more complex glycans on uncleaved gp140 (PMID 26051934). This may be just a typo that they meant complex not high mannose glycans. I think this is correct in the main text.”*

Yes. The reviewer is correct. There was a mistake in the previous response letter. However, as noted by the reviewer, the manuscript contains the correctly referenced finding that SOSIP or membrane versions of Env, contain a higher proportion of mannose glycans than the uncleaved gp140.

*“It is good that some previous papers on studying glycans on uncleaved gp140 have been cited in the discussion, but these also need to be covered in the introduction. Glycopeptide mass spec has been done by Crispin, Desaire and others on uncleaved gp140s and I think that is a key part of the introduction missing. Acknowledging this is OK, because the work here is still justified by comparing the uncleaved trimer production from two cell lines, one of which is FDA approved (CHO) and that the gp140 is in clinical trial. So, I am happy that these references were added, but they need to be said earlier to provide context.”*

We have included in the introduction (line 93) the following sentence: “From these detailed glycosylation studies it was concluded that the gp120 portion of the SOSIP trimer contains more high-mannose glycans than the gp41 portion, and that those trimers made in CHO-K1 cells contain a higher level of complex glycosylation than the cleaved trimers produced in 293 cells [45].

*“Line 94: the uncleaved gp140 is partly trimer, but also a mix of dimer and other not purified, so I think you need to drop the trimer on line 94.”*

The word “trimer” has been substituted for “Env constructs”.

**PATRONO CON IGUALDAD DE OPORTUNIDAD EN EL EMPLEO M/M/V/I**

**EQUAL EMPLOYMENT OPPORTUNITY EMPLOYER M/M/V/H**

**PO Box 365067, SAN JUAN, PUERTO RICO 00936-5067**

**PHONE (787) 758-7090, 758-2525 EXTS. 1601, 1602 FAX (787)274-8724**
